# Supplementary material for: Genome-scale requirements for dynein-based trafficking revealed by a high-content arrayed CRISPR screen
Source: bioRxiv. 2023 Mar 1:2023.03.01.530592. Preprint. [Version 1] doi: 10.1101/2023.03.01.530592 (PMC10002790; doi:10.1101/2023.03.01.530592)
Supplement: Supplement 13 [file media-13.pdf]

Supplementary table 11. Primary antibodies used for immunofluorescence

Antibody target	Host	Catalog number	Vendor	Dilution
HA	Rabbit	3724	Cell Signaling Technology	1:500
HA	Mouse	2367	Cell Signaling Technology	1:500
α-Tubulin	Mouse	T9026	Sigma Aldrich	1:3000
α-Tubulin	Rat	MCA77G	Bio-Rad	1:3000
LIS1	Mouse	H00005048-M03	Novus	1:1000
DYNC1H1	Rabbit	12345-1-AP	Proteintech	1:1000
DYNC1I2	Rabbit	HPA053987	Atlas	1:500
EEA1	Rabbit	3288	Cell Signaling Technology	1:3000
TGN46	Sheep	AHP500G	Bio-Rad	1:3000
γ-Tubulin	Mouse	T5326	Sigma Aldrich	1:1000
LAMP1	Rabbit	9091	Cell Signaling Technology	1:3000
SUGP1	Rabbit	HPA004890	Atlas	1:1000
PPIB	Rabbit	ab16045	Abcam	1:1000
DCTN1	Mouse	612708	BD	1:1000
GOLGA3	Rabbit	21193-1-AP	Proteintech	1:500
TARDBP	Rabbit	12892-1-AP	Proteintech	1:1000
V5	Rabbit	13202	Cell Signaling Technology	1:1000
V5-AF647	Mouse	451098	Thermo Fisher	1:1000
FLAG	Mouse	TA50011-100	Origene	1:1000
