# Supplementary material for: Genome-scale requirements for dynein-based trafficking revealed by a high-content arrayed CRISPR screen
Source: bioRxiv. 2023 Mar 1:2023.03.01.530592. Preprint. [Version 1] doi: 10.1101/2023.03.01.530592 (PMC10002790; doi:10.1101/2023.03.01.530592)
Supplement: Supplement 14 [file media-14.pdf]

Supplementary table 12. Secondary antibodies used for immunofluorescence

Antibody	Catalog number	Vendor	Dilution
Donkey anti-Mouse IgG (H+L) Highly Cross-Adsorbed Secondary Antibody, Alexa Fluor 488	A-21202	Thermo Fisher	1:500
Donkey anti-Rabbit IgG (H+L) Highly Cross-Adsorbed Secondary Antibody, Alexa Fluor 488	A-21206	Thermo Fisher	1:500
Goat anti-Mouse IgG (H+L) Highly Cross-Adsorbed Secondary Antibody, Alexa Fluor 568	A-11031	Thermo Fisher	1:500
Donkey anti-Rabbit IgG (H+L) Highly Cross-Adsorbed Secondary Antibody, Alexa Fluor 568	A10042	Thermo Fisher	1:500
Donkey anti-Mouse IgG (H+L) Highly Cross-Adsorbed Secondary Antibody, Alexa Fluor 647	A-31571	Thermo Fisher	1:500
Donkey anti-Rabbit IgG (H+L) Highly Cross-Adsorbed Secondary Antibody, Alexa Fluor 647	A-31573	Thermo Fisher	1:500
Donkey anti-Rat IgG (H+L) Highly Cross-Adsorbed Secondary Antibody, Alexa Fluor 647	A78947	Thermo Fisher	1:500
Donkey anti-Sheep IgG (H+L) Cross-Adsorbed Secondary Antibody, Alexa Fluor 647	A-21448	Thermo Fisher	1:500
Chromeo 494 Goat anti-mouse IgG	15032	ActiveMotif	1:300
